# The emerging role of IL‐38 in diseases: A comprehensive review

**DOI:** 10.1002/iid3.991

**Published:** 2023-08-28

**Authors:** Weijun Chen, Shuangyun Xi, Yong Ke, Yinlei Lei

**Affiliations:** ^1^ Center of Forensic Expertise Affiliated Hospital of Zunyi Medical University Zunyi Guizhou China; ^2^ School of Forensic Medcine Zunyi Medical University Zunyi Guizhou China

**Keywords:** cardiovascular and cerebrovascular diseases, interleukin‐38, lung diseases, signal path, viral infectious diseasesautoimmune diseases

## Abstract

**Introduction:**

Interleukin‐38 (IL‐38) is a new type of anti‐inflammatory cytokine, which is mainly expressed in the immunity‐related organs and is involved in various diseases including cardiovascular and cerebrovascular diseases, lung diseases, viral infectious diseases and autoimmune diseases.

**Aim:**

This review aims to detail the biological function, receptors and signaling of IL‐38, which highlights its therapeutic potential in related diseases.

**Conclusion:**

This article provides a comprehensive review of the association between interleukin‐38 and related diseases, using interleukin‐38 as a keyword and searching the relevant literature through Pubmed and Web of science up to July 2023.

## Graphical abstract

This review aims to detail the biological function of IL‐38, receptors and signaling of IL‐38, and the role of IL‐38 in related diseases, which highlights the therapeutic potential of IL‐38 in these diseases

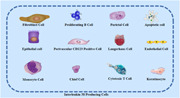

## 1 OVERVIEW OF INTERLEUKIN‐38 (IL‐38)

**Figure 1:**
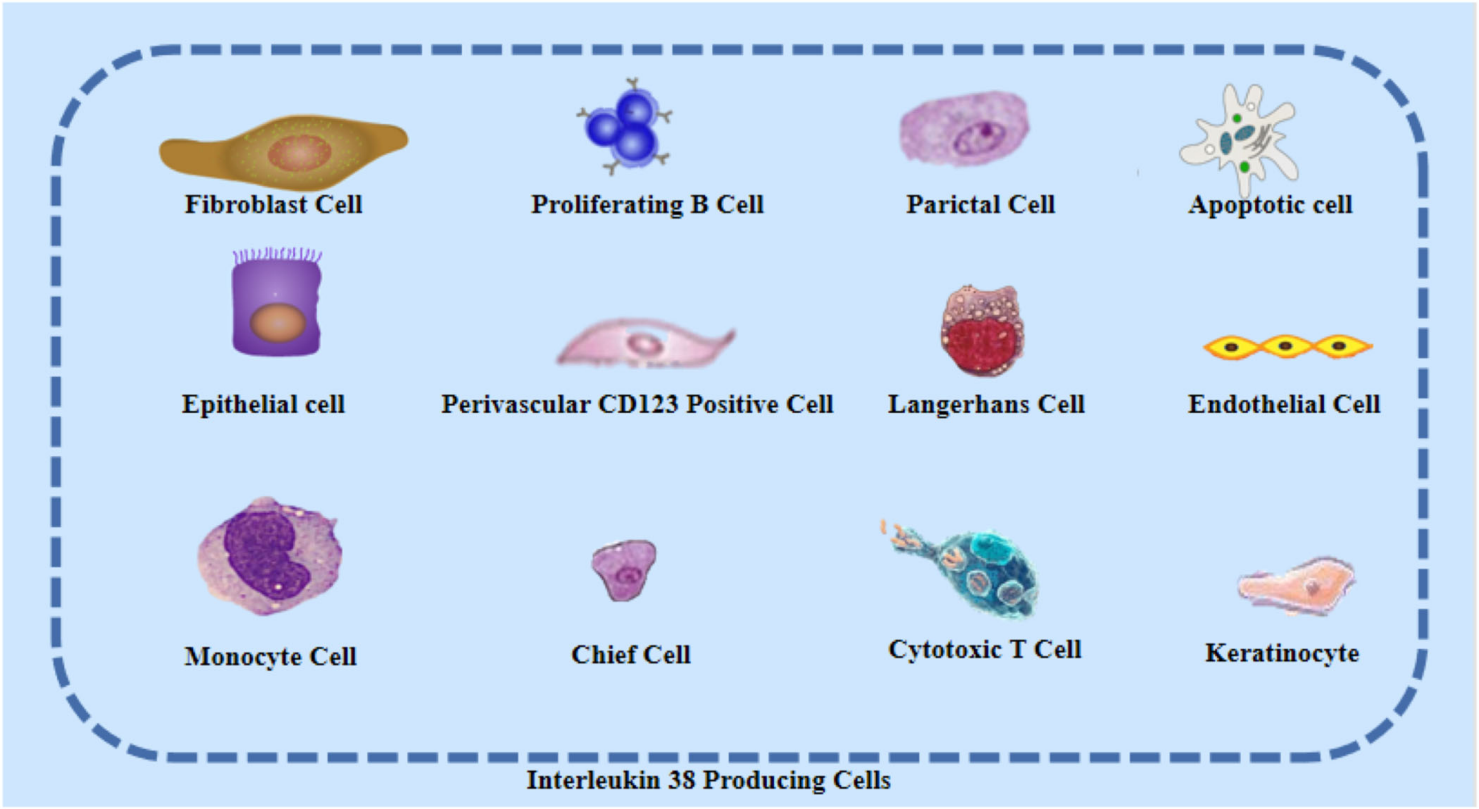
Production of IL‐38: IL‐38 is highly expressed in immune tissues and less in inactive immune tissues. IL‐38 can be secreted by fibroblast cell, proliferating B cell, parictal cell, apoptotic cell, epithelial cell, perivascular CD123 positive cell, Langerhans cell, endothelial cell, monocyte cell, chief cell, cytotoxic T cell, keratinocyte, etc. IL‐38, interleukin‐38.

The IL‐1 family includes 11 cytokine members, in which IL‐1α, IL‐1β, IL‐18, IL‐33, IL‐36α, IL‐36β, and IL‐36γ have agonist activity, IL‐1Ra and IL‐36Ra are antagonists of receptors, and IL‐37 and IL‐38 exert anti‐inflammatory functions.^1^, ^2^ IL‐38 was first discovered in 2001 and was called IL‐1HY2 or IL‐1F10 at that time. It belongs to IL‐36 subfamily that contains IL‐36α, IL‐36β, IL‐36γ, and IL‐36Ra and can interact with a variety of receptors thereby inhibiting the expression of proinflammatory factors.^3^, ^4^ IL‐38 protein has a molecular weight of about 17 kDa, and it has a homological structure with IL‐1Ra (37%) and IL‐36Ra (41%).^2^, ^3^ IL‐38 is mainly expressed in organs that are involved in immune responses, and its expression is relatively low in tissues without specific role in immunity.^5^, ^6^, ^7^ IL‐38 is secreted by epithelial cells, monocytes, macrophages, and immune cells (Figure 1). The precursor of IL‐38 requires N‐terminal cleavage, which can bind to receptors and recruit downstream factors, thereby exerting biological activities. Inflammatory response plays an important role in human physiological and pathological processes, and the cytokine IL‐38 has been identified to have a protective role in diseases.^8^, ^9^, ^10^ Therefore, this review searched public databases for relevant publications of IL‐38, aiming to summarize the role and mechanism of IL‐38 in various system diseases and to discuss the future directions of prevention and treatment.

## 2 RECEPTORS OF IL‐38

The current IL‐1 family receptors include IL‐1R1 (IL‐1RI), IL‐1R2 (IL‐1RII), IL‐1RAcP, ST2, IL‐18Rα, IL‐1Rrp2 (IL‐36R), IL‐18Rβ, TIR8 (SIGIRR), TIGIRR‐2, and TIGIRR‐1. Among them, IL‐1R1, IL‐36R, and IL‐1RAPL1 (also known as IL1R9, TIGIRR‐2) are main receptors of IL‐38.^4^, ^11^, ^12^ IL‐1R1 is critical for innate immune response and mediates proinflammatory effects when it binds with inflammatory cytokines. IL‐1Ra can interfere with the recruitment and binding of IL‐1RAcP to IL1R1, which suppresses the signaling of IL‐1R1. Similar to IL1Ra, IL‐36Ra blocks the signaling of IL‐36R via recruiting inhibitory single IL‐1 receptors such as SIGIRR. Since IL‐38, IL‐1Ra and IL‐36Ra have homological structure, it is suspected that IL‐38 has similar function to the two cytokines. Therefore, IL‐38 may recruit inhibitory IL‐1 receptors to suppress IL‐36R signaling. However, whether IL‐38 can recruit SIGIRR has not been reported. IL‐38 was found to have a higher affinity to IL‐36R compared with IL‐36Ra and IL‐1R1,^13^ however, its affinity to IL‐1R1 was the weakest compared to IL‐1Ra and IL‐1beta (93 nM vs. 38 nM vs. 21 nM).^3^ Meanwhile, IL1RAPL1 is another receptor for IL‐38. IL1RAPL1 is associated with cerebellar development, intellectual disability, and cognitive deficits. IL‐38 that is produced by apoptotic cells has a strong affinity with IL1RAPL1 to limit the activation of inflammatory macrophages and Th17 cells by blocking the IL1RAPL1 signaling pathway.^14^ Therefore, IL1RAPL1, IL‐1R1 and IL‐36R are main receptors of IL‐38, among which IL‐36R has the strongest binding ability.

## 3 SIGNALING OF IL‐38

**Figure 2:**
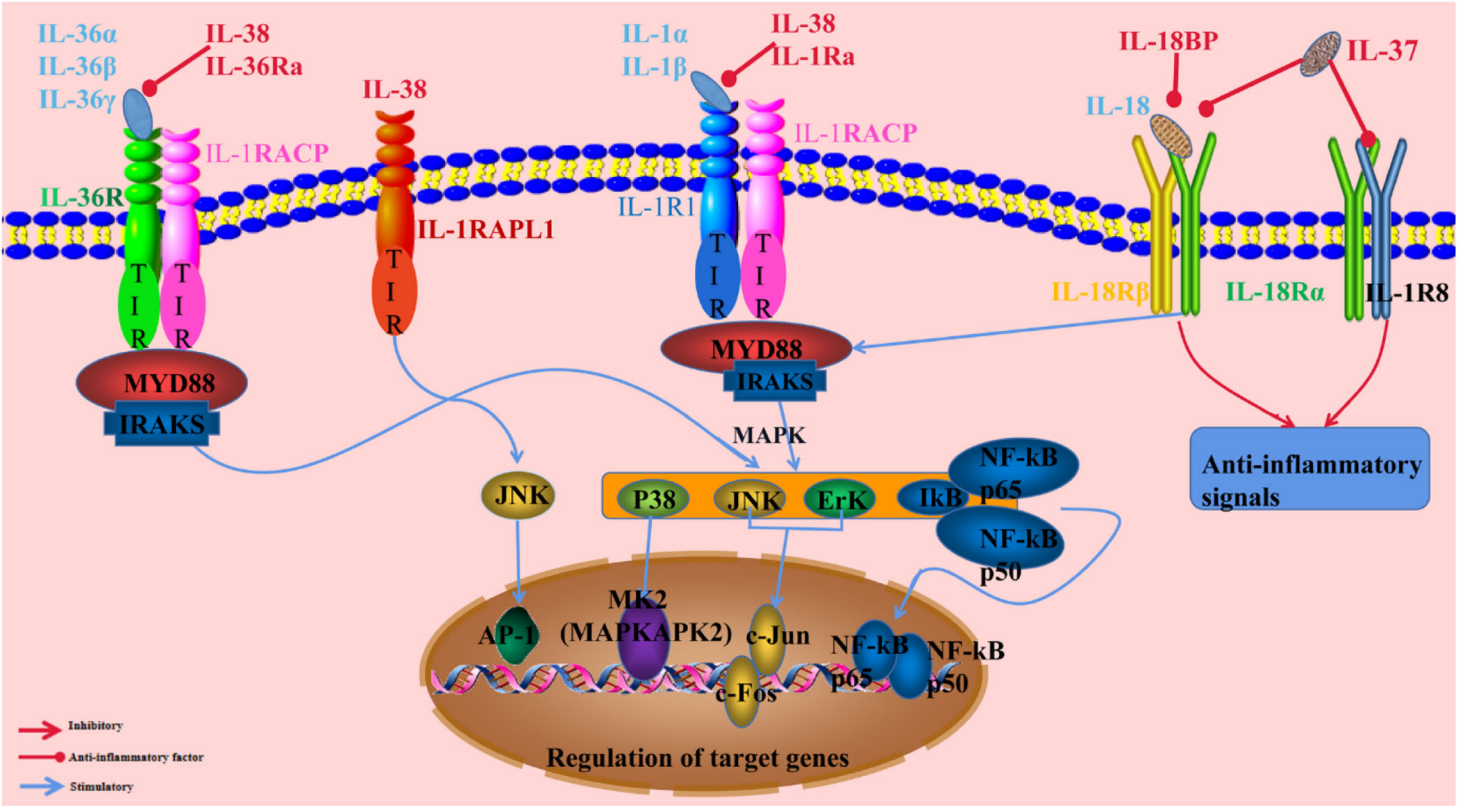
Illustration of IL‐38 signaling pathway: IL‐38 regulates immune and inflammatory responses by binding to its receptors and activating downstream signaling. For instance, IL‐38 competitively binds to the receptor IL36R along with interleukins 36α, 36γ, and 36β. Moreover, IL‐38 binds to the receptor IL‐1R1 along with IL‐1α and IL‐1β, thus inhibiting the recruitment of IL‐1RAcP and MyD88 and the activation of downstream signaling pathways such as NF‐κB, ERK, JNK, P38, etc. IL‐18 binds to IL‐18Rα and IL‐18Rβ to recruit MyD88 and activate downstream signaling pathways. IL‐18 binding protein (IL‐18BP) and IL37 competes with IL‐18 for binding to IL‐18Rα, which prevents the binding of IL‐18 and blocks the signaling. IL‐38, interleukin‐38; NF‐κB, nuclear factor‐κB.

As above mentioned (Figure 2), the structure of IL‐38 is similar to IL‐36Ra, which can inhibit signaling of IL‐36 via recruiting IL‐1RAcP,^14^ indicating that IL‐38 may exert similar effects via this mechanism. Meanwhile, IL‐38 has a homologic structure compared with IL‐1Ra, which can bind to IL‐1RAcP to form a heterotrimeric complex, resulting in TIR domain aggregation and MyD88‐binding protein elevation. Hence, IL‐38 can prevent the recruitment of IL‐1RAcP to exert inhibitory signaling. Meanwhile, IL‐1Ra was an antagonist of IL‐1R family including ST2/T1, SIGIRR, TIGIRR1/2.^15^ Similar to IL‐1Ra, IL‐38 was reported to bind to IL1RAPL1, also termed TIGIRR‐2, to inactivate JNK phosphorylation and AP1 activation, thereby inhibiting cytokine production in macrophages.

## 4 ROLE OF LL‐38 IN RELATED DISEASES

### 4.1 The role of lL‐38 in pulmonary diseases

IL‐38 is closely related to pulmonary diseases. Studies have shown that inflammatory cytokines play important roles in acute respiratory distress syndrome (ARDS).^16^, ^17^ Chai et al.^18^ found that patients with ARDS had a higher level of IL‐38. IL‐38 could inhibit Th17 cell differentiation to prevent the occurrence of ARDS, therefore, IL‐38 was a potential strategy for treating ARDS.^19^ Allergic asthma is a common inflammatory disease, where IL‐38 was demonstrated to exert anti‐inflammatory effects via regulating P38, STAT1, STAT3, ERK, and nuclear factor‐κB (NF‐κB) signaling pathways. Interstitial lung disease is a diffuse disease that affects the lung interstitium, alveoli, or bronchioles. Tominaga et al.^20^ found that IL‐38 could increase the expression of anti‐fibrotic factor interferon‐γ, reduce the expression of tumor necrosis factor‐α (TNF‐α), and improve pulmonary fibrosis in Bleomycin (BLM) induced pulmonary fibrosis. Sun et al.^21^ found that in pneumonia patients, the serum level of IL‐38 was elevated and its level was negatively associated with clinical inflammatory indicators. Moreover, the recombinant IL‐38 significantly was able to reduce the level of inflammatory cytokines and the adhesion molecule ICAM‐1.^21^ Therefore, IL‐38 acts as anti‐inflammatory and anti‐fibrotic factors to ameliorate pulmonary diseases.

### 4.2 The role of IL‐38 in central nervous system diseases

Inflammatory demyelinating diseases are autoimmune diseases in presented with demyelination of the central nervous system and infiltration of perivascular inflammatory cells into small vessels. They include neuromyelitis optica spectrum disorder (NMOSD), transverse myelitis, optic neuritis, etc, where IL‐38 play a crucial role in their pathogenesis. In NMOSD, IL‐17 produced by Th17 cells contributes to disease progression, which can be inhibited by IL‐38 via reducing the secretion of chemokines. Autism spectrum disorder (ASD) is a developmental disability with unclear pathogenic mechanisms. Irene Tsilioni et al showed that neurotensin (NT) stimulated microglia to secrete IL‐1β and CXCL8 in ASD and this process could be inhibited by IL‐38, suggesting that IL‐38 could be a potential therapy for the treatment of ASD.^22^, ^23^ As a neurodegenerative disorder, Alzheimer's disease (AD) is characterized by mental decline, behavioral impairment, and cognitive impairment.^24^ The neuroinflammation mediated by microglia is suggested to be involved in AD development.^25^, ^26^ Microglia expresses receptors such as toll‐like receptors to activate NLRP3 inflammasome to inflammation. The binding of amyloid‐beta to these receptors promotes the release of inflammatory factors that can promote AD progression.^27^, ^28^ IL‐38 can significantly downregulate the expression of IL‐1β and TNF‐α released by lipopolysaccharide‐stimulated macrophages,^29^ indicating that IL‐38 may exert therapeutic effects on AD via suppressing neuroinflammation, however, the underlying mechanism needs to be further investigated.

Ischemic stroke refers to insufficient blood supply to the brain. Strokes are classified into ischemic stroke and hemorrhagic one. Conventional treatment for ischemic stroke includes directly intravenous injection of recombinant tissue plasminogen activator (tPA) to the blocked blood vessel. tPA promotes the conversion of plasminogen to plasmin, which can degrade and dissolve fibrin clots. Zare Rafie et al.^30^ showed that after tPA treatment in ischemic stroke patients, the serum level of IL‐38 significantly increased in 24 h, and its level was associated with the 3‐month prognosis of patients. Hence, IL‐38 may be an early and reliable marker for predicting the prognosis of ischemic stroke patients.^31^

### 4.3 The relationship between IL‐38 and cardiovascular diseases

Cardiovascular disease (CVD) refers to a group of diseases with high mortality rate.^32^ Abdominal aortic aneurysm (AAA) refers to pathologically dilated arterial wall of the lower abdomen of the aorta. IL‐38 may protect against AAA formation by regulating the accumulation and phenotype of macrophage through IL1RL2‐p38 pathway, therefore, it is speculated that IL‐38 may be a new therapeutic modality for the treatment of AAA.^33^ Atherosclerosis is the main pathogenic factor for CVDs and originated from vascular endothelial injury. IL‐38 can inhibit endothelial cell proliferation and migration via reducing the expression of angiogenic factors. Also, IL‐38 can decrease the expression of IL‐8 and TNF‐α to limit the activation of angiogenesis.^34^, ^35^, ^36^, ^37^, ^38^ Wei et al.^39^ showed that IL‐38 inhibited the inflammatory response in the infarcted heart, and inflammatory cardiomyocytes inhibited apoptosis and alleviated ventricular remodeling through the release of IL‐38.^40^ Although reperfusion therapy was an effective method to address coronary occlusion, excessive myocardial ischemia‐reperfusion injury remains existed. IL‐38 could inhibit the activation of NLRP3 inflammasome, thereby suppressing macrophage‐mediated inflammation and reducing apoptosis in myocardium.^41^ Obesity and diabetes are key risk factors for CVDs. IL‐38 is able to inhibit preadipocyte differentiation by promoting GATA‐3 expression, reduce triglyceride synthesis and decrease adipocyte size to ameliorate obesity, and ameliorate insulin resistance.^19^ In addition, IL‐38 can reduce the release of IL‐1β, IL‐6, and MCP‐1 and the levels of TC, TG, and LDL, thereby improving lipid and glucose metabolism and reducing the risk of CVDs.^42^, ^43^ de Graaf et al.^44^ reported that compared with healthy persons, serum IL‐38 level was significantly lower in overweight persons with high risk of CVDs, and its level was lowest in patients with metabolic disorders. Moreover, the level of IL‐38 was negatively correlated with that of C‐reactive protein, IL‐6 and IL‐1Ra in healthy participants. Also, serum IL‐38 level and IL‐38 expression were significantly increased in patients with acute myocardial infarction. Compared with the control group, IL‐38 level in the reperfusion‐treated patients was significantly reduced and returned to normal after 7 days, which was opposite to the expression pattern of cardiac troponin I (cTnI), a marker of myocardial infarction.^45^ Hence, IL‐38 is a potential biomarker in acute myocardial infarction patients. The et al.^46^ found that IL‐38 inhibited osteogenic activity in the aortic valve by inhibiting the NLRP3 inflammasome and caspase‐1, indicating that IL‐38 had the potential to prevent aortic valve calcification. It has been shown that IL‐38 is an independently factor of stroke and death in patients with atrial fibrillation.^47^, ^48^ In addition, a predictive model composed of IL‐38, NT‐proBNP and CHA2DS2‐VASc had a potent accuracy in predicting atrial fibrillation‐related mortality and patients’ prognoses. Consequently, IL‐38 can be used as a potential biomarker and a therapeutic target for CVDs.

### 4.4 The relationship between IL‐38 and viral infectious diseases

The role of IL‐38 in infectious diseases has attracted widespread attention. Gao et al.^49^ revealed that the level of IL‐38 and IL‐36α was significantly elevated in patients with influenza and COVID‐19, in which IL‐38 level was negatively and IL‐36α was positively associated with infection severity. Wang et al.^50^ showed elevated level of IL‐38 in patients with chronic hepatitis B, suggesting that IL‐38 could be used as a biomarker for liver hepatitis infection and liver injury.^51^ Fazeli et al.^52^ reported that the level of IL‐38 was higher in healthy subjects than treated HCV‐infected patients, and HCV‐infected patients with higher levels of IL‐38 indicated less liver damage. In sepsis mice model, the level of IL‐38 increased and IL‐38 could prolong the survival of mice.^53^ Xue et al.^54^ demonstrated that the neutralization of IL‐38 exacerbated coxsackievirus B3‐induced viral myocarditis (AVMC) in mice, possibly due to a Th1/Th17 cell imbalance and elevated virus replication. These findings indicate that IL‐38 plays a protective role during pathogen infection and is a potential therapeutic target for infectious diseases.

### 4.5 The role IL‐38 in autoimmune diseases

IL‐38 is reported to regulate the development of autoimmune diseases by inactivating immune cells and inflammatory responses through various mechanisms.^55^, ^56^ For example, IL‐38 can inhibit the release of inflammatory cytokines and chemotaxis to inactivate inflammatory cells. Meanwhile, IL‐38 promotes the activity of regulatory T cells (Treg) to suppress autoimmune responses. In patients with systemic lupus erythematosus (SLE), serum levels of IL‐38 are significantly elevated and its high levels are associated with the remission of SLE‐related symptoms, including proteinuria, leukocyturia and skin lesions, and it has also been reported that IL‐38 is involved in the progression of SLE by regulating the NF‐κB signaling pathway,^57^, ^58^, ^59^ suggesting that IL‐38 may play a play a protective role in SLE. In addition, the role of IL‐38 in psoriasis has been extensively explored, with IL‐38 expression levels is correlated with the severity of psoriasis.^11^, ^60^, ^61^, ^62^, ^63^, ^64^ Cytokines secreted by Th1 and Th17 cells contribute to the pathogenesis of psoriasis, and IL‐38 is involved in regulating the secretion of IL‐17 and IL‐22 by Th17 cells.^65^, ^66^ Moreover, Th17 cells are involved in the progression of various autoimmune diseases,^60^, ^67^, ^68^ such as rheumatoid arthritis,^26^, ^36^ primary Sjögren's syndrome,^69^, ^70^ inflammatory bowel disease,^8^ ankylosing spondylitis,^71^ allergic rhinitis,^72^ glaucoma,^73^ septic dermatitis, multiple sclerosis, and autoimmune thyroid disease,^74^, ^75^, ^76^, ^77^, ^78^, ^79^, ^80^ suggesting that IL‐38 may play a key role in autoimmune diseases. However, its mechanism remains largely unclear. Therefore, additional research is needed to explore the role of IL‐38‐related signaling in autoimmune diseases and find proper IL‐38 modulators to be potential therapeutics for autoimmune diseases.

### 4.6 The role of lL‐38 in cancer

In recent years, IL‐38 has attracted a lot of attention in tumor immunology. Colorectal cancer (CRC), a malignant tumor derived from colonic and rectal mucosal epithelial, is one of the common malignancies worldwide. IL‐38 was lowly expressed in CRC tissue compared to adjacent colon tissue. Its expression was associated with TNM stages, tumor size, tumor infiltration, and tumor differentiation. Moreover, receiver operating characteristic analysis showed that IL‐38 was a potential biomarker for diagnosing CRC.^81^ Meanwhile, IL‐38 could increase cell apoptosis and inhibit the migration and proliferation of CRC cellsb.^82^ These studies indicated that IL‐38 played a protective role in CRC progression and could be used as a biomarker for CRC diagnosis and to predict the prognosis of CRC patients. In addition, IL‐38 expression was high in various cancers such as lung, esophageal, and breast cancers, etc.^83^ IL‐38 level was associated with high tumor grade, late T stage, late N stage, advanced tumor stage, and pleural and vascular metastasis.^84^ Zhou et al.^85^ revealed that IL‐38 was involved in regulating epidermal cell proliferation and the pro‐tumor microenvironment through the IL‐1Rrp2/JNK signaling pathway. The blockade of IL‐38 promote immune infiltration, the generation of tumor‐specific memory, and the elimination of tumor growth.^86^ However, the crosstalk mechanism between IL‐38 and tumor microenvironment in different cancers requires further investigation. Nevertheless, IL‐38 can be used as a potential target for cancer diagnosis, treatment and prognosis prediction.

## 5 CONCLUSION

As a novel cytokine, IL‐38 is involved in the progression of different diseases. Although IL‐38 is suggested to play a protective role in most diseases, its regulatory mechanism and related signaling remain largely unknown. Meanwhile, IL‐38 has the potential to be a therapeutic method for these diseases. Different forms of IL‐38 have different biological functions. For example, truncated IL‐38 exerts anti‐inflammatory effects, while full‐length IL‐38 is associated with the level of IL‐6 and CXCL8 in blood. Therefore, a better understanding of molecular mechanisms of different IL‐38 forms will facilitate its clinical translation.

## AUTHOR CONTRIBUTIONS

**Weijun Chen**: conceptualization; data curation; formal analysis; writing—original draft. **Shuangyun Xi**: formal analysis; investigation. **Yong Ke**: funding acquisition; investigation; methodology. **Yinlei Lei**: data curation; formal analysis.

## CONFLICT OF INTEREST STATEMENT

The authors declare no conflicts of interest.

## Data Availability

The data that support the findings of this study are available from the corresponding author upon reasonable request.
