# Correction to “The emerging role of IL‐38 in diseases: A comprehensive review”

**DOI:** 10.1002/iid3.1287

**Published:** 2024-05-29

Chen W, Xi S, Ke Y, Lei Y. The emerging role of IL‐38 in diseases: A comprehensive review. Immun Inflamm Dis. 2023;11(8):e991. https://doi.org/10.1002/iid3.991

The original article included images from Fazeli et al. 52 for which the authors did not have permission for re‐use. Permission has been retrospectively obtained from the Fazeli et al. and the copyright holder.

The caption for Figure 1 should now read: Production of IL‐38: IL‐38 is highly expressed in immune tissues and less in inactive immune tissues. IL‐38 can be secreted by fibroblast cell, proliferating B cell, parictal cell, apoptotic cell, epithelial cell, perivascular CD123 positive cell, Langerhans cell, endothelial cell, monocyte cell, chief cell, cytotoxic T cell, keratinocyte, etc. IL‐38, interleukin‐38. The following images: [Diagrams of three cell models in Figure 2: Chief cell, prevascular CD123‐cell, parictal cell] were adapted with permission from Fazeli et al. (52), Copyright (2022).

We apologize for this error.

52. Fazeli P, Saeidnia M, Erfani M, Kalani M. An overview of the biological and multifunctional roles of IL‐38 in different infectious diseases and COVID‐19. Immunol Res. 2022;70(3):316‐324.

